# Supplementary figures and images for: Repeated rebiopsy for detection of EGFR T790M mutation in patients with advanced-stage lung adenocarcinoma: Associated factors and treatment outcomes of Osimertinib
Source: PLoS One. 2024 Sep 19;19(9):e0310079. doi: 10.1371/journal.pone.0310079 (PMC11412630; doi:10.1371/journal.pone.0310079)

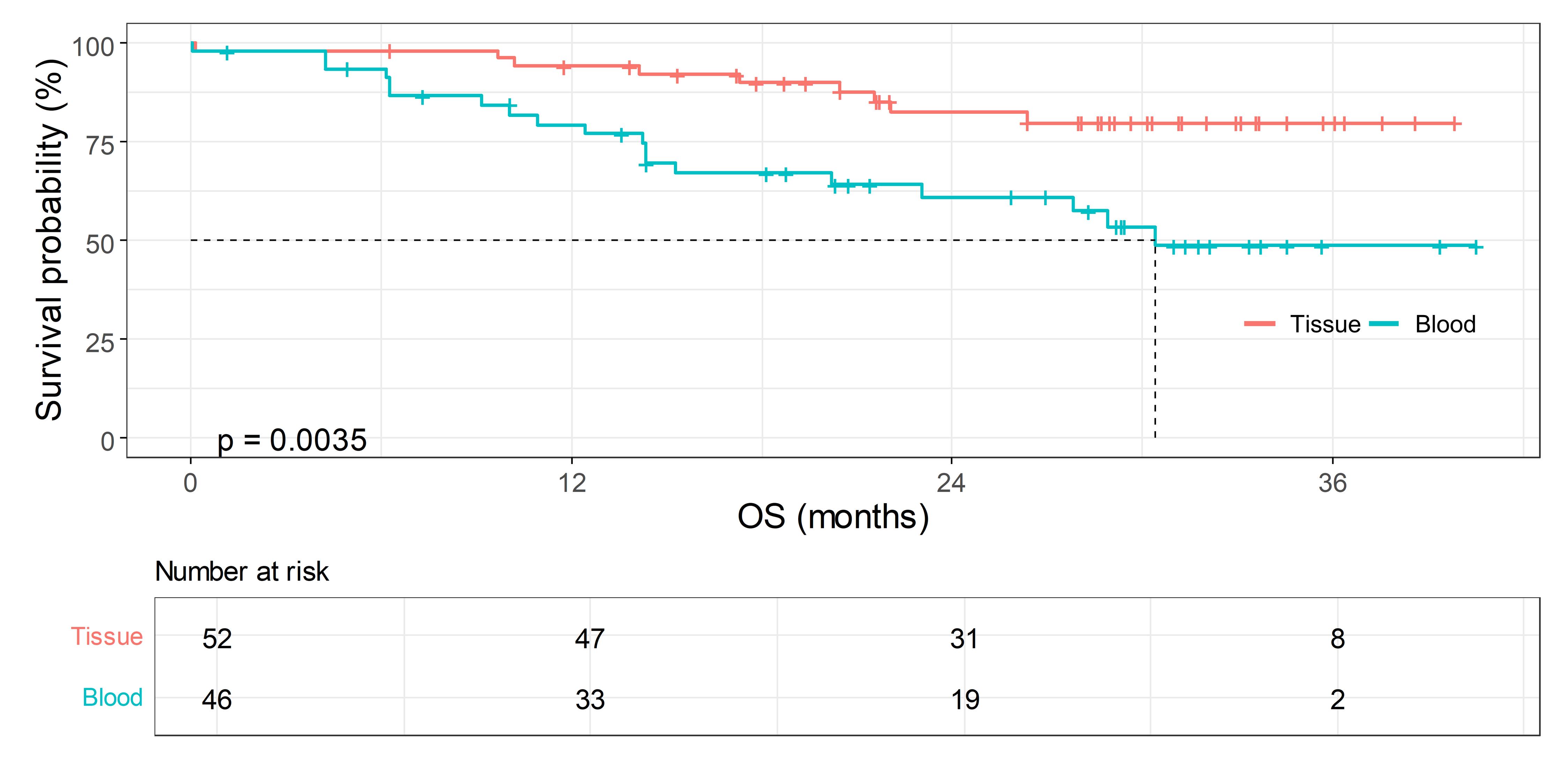

Supplement: S1 Fig — OS, overall survival. *Patients who were diagnosed with stage 3, 4A, or 4B at their first diagnosis were analyzed. (TIF) [file pone.0310079.s001.tif]
