# Supplementary material for: Repeated rebiopsy for detection of EGFR T790M mutation in patients with advanced-stage lung adenocarcinoma: Associated factors and treatment outcomes of Osimertinib
Source: PLoS One. 2024 Sep 19;19(9):e0310079. doi: 10.1371/journal.pone.0310079 (PMC11412630; doi:10.1371/journal.pone.0310079)
Supplement: S2 Table — (DOCX) [file pone.0310079.s003.docx]

**S1 Table.** Progression-free survival (months) according to the number of rebiopsies and methods for confirmation of T790M mutation in patients initially diagnosed with advanced disease

|  | **1-year PFS rate** | **Median** | **95% CI** | **p** |
| --- | --- | --- | --- | --- |
| **Number of rebiopsies** |  |  |  | 0.32 |
| One | 50.7% | 12.2 | 8.3–16.1 |  |
| Two or more | 35.1% | 9.4 | 6.1–12.7 |  |
| **Confirmation of T790M** |  |  |  | 0.825 |
| Tissue | 49.4% | 12.0 | 7.4–16.6 |  |
| Plasma | 43.4% | 10.8 | 7.4–14.3 |  |
| **Confirmation of T790M** |  |  |  | 0.751 |
| Tissue, first | 59.3% | 16.6 | 2.9–30.3 |  |
| Plasma, first | 45.3% | 10.8 | 6.2–15.4 |  |
| Tissue, second or more | 38.3% | 9.4 | 5.4–13.3 |  |
| Plasma, second or more | 25.0% | 5.6 | NA–12.9 |  |

PFS, progression free survival; CI, confidence interval; NA, not available; NR, not reached
