# Supplementary material for: Repeated rebiopsy for detection of EGFR T790M mutation in patients with advanced-stage lung adenocarcinoma: Associated factors and treatment outcomes of Osimertinib
Source: PLoS One. 2024 Sep 19;19(9):e0310079. doi: 10.1371/journal.pone.0310079 (PMC11412630; doi:10.1371/journal.pone.0310079)
Supplement: S3 Table — (DOCX) [file pone.0310079.s004.docx]

**S2 Table.** Overall survival (months) according to the number of rebiopsies and methods for confirmation of T790M mutation in patients initially diagnosed with advanced disease

|  | **2-year OS rate** | **Median** | **95% CI** | **p** |
| --- | --- | --- | --- | --- |
| **Number of rebiopsies** |  |  |  | 0.57 |
| One | 70.5% | NR | NA–NA |  |
| Two or more | 72.2% | NR | NA–NA |  |
| **Confirmation of T790M** |  |  |  | 0.0035 |
| Tissue | 82.5% | NR | NA–NA |  |
| Plasma | 61.0% | 30.4 | 23.1–NA |  |
| **Confirmation of T790M** |  |  |  | 0.02 |
| Tissue, first | 84.3% | NR | NA–NA |  |
| Plasma, first | 62.9% | 30.4 | 23.1–NA |  |
| Tissue, second or more | 80.8% | NR | NA–NA |  |
| Plasma, second or more | 33.3% | 14.2 | 6.14–NA |  |

OS, overall survival; CI, confidence interval; NA, not available; NR, not reached
