# Supplementary material for: Repeated rebiopsy for detection of EGFR T790M mutation in patients with advanced-stage lung adenocarcinoma: Associated factors and treatment outcomes of Osimertinib
Source: PLoS One. 2024 Sep 19;19(9):e0310079. doi: 10.1371/journal.pone.0310079 (PMC11412630; doi:10.1371/journal.pone.0310079)
Supplement: S4 Table — (DOCX) [file pone.0310079.s005.docx]

**S3 Table.** Patient characteristics according to sites of T790M confirmation

|  | **Tissue**  **(n = 72)** | **Plasma**  **(n = 65)** | **p** |
| --- | --- | --- | --- |
| **Age, years** | 64.93 ± 9.71 | 67.42 ± 10.63 | 0.155 |
| **Female sex** | 39 (54.2) | 38 (58.5) | 0.739 |
| **Body mass index, kg/m^2^** | 23.84 ± 3.38 | 22.74 ± 3.65 | 0.069 |
| **Ever smokers** | 25 (34.7) | 25 (38.5) | 0.782 |
| **Charlson’s comorbidity index** | 10 (13.9) | 12 (18.5) | 0.621 |
| **Stage 4B** | 26 (38.2) | 46 (71.9) | <0.001 |
| **Number of metastatic organs** |  |  | 0.156 |
| **0–1** | 31 (43.1) | 21 (32.3) |  |
| **2–3** | 35 (48.6) | 32 (49.2) |  |
| **≥4** | 6 (8.3) | 12 (18.5) |  |
| **Brain metastasis** | 18 (26.5) | 30 (46.9) | 0.024 |
| **Liver metastasis** | 5 (7.4) | 12 (18.8) | 0.09 |
| **Bone metastasis** | 17 (25.0) | 37 (57.8) | <0.001 |
| **Lung-to-lung metastasis** | 38 (55.9) | 31 (48.4) | 0.496 |
| **Pleural metastasis** | 39 (57.4) | 23 (35.9) | 0.022 |

Data are presented as mean ± standard deviation for continuous variables and n (%) for categorical variables.
