# Supplementary material for: Repeated rebiopsy for detection of EGFR T790M mutation in patients with advanced-stage lung adenocarcinoma: Associated factors and treatment outcomes of Osimertinib
Source: PLoS One. 2024 Sep 19;19(9):e0310079. doi: 10.1371/journal.pone.0310079 (PMC11412630; doi:10.1371/journal.pone.0310079)
Supplement: Attachment — Submitted filename: Response to Reviewers_revised_240727.docx [file pone.0310079.s006.docx]

**Reviewer 1**

**Comment 1**: It would have been helpful to give some information about the entire patient population: 100% had T790M after max 3 biopsies, but how many received biopsies (i.e. how many other patients had all biopsies negative) in the same time period?

**Response 1**: Thank you for the valuable comment.

In response to the comments from both reviewers, we re-checked the entire patient population who received rebiopsies during the study period. Therefore, we have also revised the patient selection process. In brief, a total of 443 patients received rebiopsies, with the cumulative rate of T790M detection increasing as follows: 14.2% after the first rebiopsy, 31.8% after the second, 38.8% after the third, 41.1% after the fourth, 41.5% after the fifth, and 42.0% after the sixth.

We summarized these results as **Fig 1** and **Table 1**. Also, we have revised the related sections: Abstract (Results), Materials and Methods, 1^st^ paragraph of Results, and 1^st^ and 3^rd^ paragraph of Discussion.

**Comment 2 & 3**: It would be important to add the type of EGFR mutation in Table 1 (del19 vs. L858R vs. other rare mutations). Regarding the analysis of survival: it would be important to also check other prognostic parameters, like the type of EGFR mutation. It is known that the type of EGFR mutation and presence of brain metastases are independent predictors of OS for these patients, which should be taken into account in the analysis, see for example https://pubmed.ncbi.nlm.nih.gov/32871455/, which could also be added in the Discussion.

**Response 2 & 3**: Thank you for the comment and sharing a valuable reference.

First, we have added numbers and percentages of EGFR mutation in **Table 2**. Second, we have re-estimated odds ratio from multivariable analyses additionally considering EGFR mutation status. Results are summarized in **Table 5**: however, in this analysis we could not find EGFR mutation as a distinguishing parameter in terms of number of rebiopsy and method for rebiopsy. Please refer to the Results section.

**Reviewer 2**

Major points:

**Comment 1 & 2**: Their study population focused on only osimertinib-administered patients. They insisted T790M detection rate on first rebiopsy of 69.9%. This is absolutely incorrect. As they mentioned, many studies reported clinical T790M detection rate on first rebiopsy of 30-50%. These previous studies consecutively examined all patients who received rebiopsy to detect T790M in their hospitals. The authors should also consecutively examine all rebiopsied patients in their institute. This is a critical problem in their study.

Similarly, T790M detection rates on second and third rebiopsies were incorrect. They excluded cases without T790M, since their study only examined T790M-positive cases.

**Response 1 & 2**: Thank you for the valuable comment.

In response to the comments from both reviewers, we have re-checked the entire patient population who received rebiopsies during the study period. Therefore, we have also revised the patient selection process. In brief, a total of 443 patients received rebiopsies, with the cumulative rate of T790M detection increasing as follows: 14.2% after the first rebiopsy, 31.8% after the second, 38.8% after the third, 41.1% after the fourth, 41.5% after the fifth, and 42.0% after the sixth.

We have summarized these results as **Fig 1** and **Table 1**. Also, we have revised the related sections: Abstract (Results), Materials and Methods, 1^st^ paragraph of Results, and 1^st^ and 3^rd^ paragraph of Discussion.

**Comment 3**: They described “The median interval between the first and second rebiopsy was 12.5 days (IQR: 8.5-21.0 days).” This is astonished. After failure of first rebiopsy, was second rebiopsy performed on the same target? or another target? Their manuscript did not show detailed information on rebiopsy targets.

**Response 3**: Thank you for the comment. The next target for rebiopsy was based on the clinician’s decision rather than on a protocolized approach. Therefore, we clarified this point in the method section.

Minor point:

**Comment 1**: EGFR should be italicized when they express a gene. (e.g., EGFR mutation)

**Response 1**: We have italicized *EGFR* when it expresses the gene.
